# The Ninth International Medical Congress

**Published:** 1885-12

**Authors:** 


### THE NINTH INTERNATIONAL MEDICAL CONGRESS.

So much has already been said and written regarding the next International Medical Congress, and the mistakes and misunderstandings attending its organization, that it is an unwelcome task to say more regarding it. Since, however, there appears to be much confusion of ideas, and, also, such an erroneous impression at home and abroad, of the history of the efforts at organization, it may lead to a better understanding of the case if the facts be given, *ab initio*, in chronological order.

During the annual meeting of the American Medical Association in Washington, D. C., in May, 1884, a desire was expressed that an invitation be extended to the Eighth International Medical Congress, about to assemble in Copenhagen, Denmark, to hold the next Triennial Congress in Washington, D. C. It was understood that in extending such invitation the American Medical Association was selected, because it represented, more fully than any other medical organization in the United

States, the whole medical profession of our country. For that reason the medical profession considered that its wish and its invitation would be regarded as more authoritatively expressed if the American Medical Association would consent to become sponsor for it, and to issue the invitation. The president of the Association, in his address, advised compliance with this expressed wish. Thereupon, that part of the president's address was referred to a committee, who reported as follows:

"Dr. J. S. Billings, of the Committee on International Medical Congress, presented the following: 'The committee to which was referred that part of the president's address relating to the proposed meeting of the International Medical Congress in the United States, in 1887, has the honor to report that it has conferred, so far as the limited time at its disposal would allow, with leading members of the Association, representing all parts of the country, and that the sentiment appears to be practically unanimous in favor of carrying out the suggestions made by the president.

"'The Committee accordingly respectfully submits the following resolutions with the recommendation that they be adopted by the Asssociation:

"'*Resolved*, 1. That a Committee of seven, of which Dr. Austin Flint, the President of this Association shall be a member, shall be appointed by the President.

"'2. It shall be the duty of this Committee to extend in behalf of the medical profession of the United States to the International Medical Congress, about to meet at Copenhagen, a cordial invitation to have the next International Medical Congress meet at Washington, D. C., in 1887.

"'*Resolved*, 2. That the Committee shall elect its own officers, and that, in case the invitation is accepted, it shall proceed to act as an Executive Committee, with full power to fix the time and to make all necessary and suitable arrangements for the meeting of such Congress, and to solicit funds for this purpose.

"'3. That the Committee shall have power to add to its membership; to perfect its organization, and that to meet the preliminary expenses of printing, postage, etc., the chairman of this Committee is authorized to draw upon the Treasurer of this Association for an amount not exceeding four hundred dollars.

"'(Signed) "'J. S. BILLINGS,
"'L. A. SAYRE,
"'I. MINIS HAYS,
"'H. F. CAMPBELL.

"'R. W. Foster not present.'

"On motion, the report was adopted."

The committee thus appointed proceeded to Copenhagen and presented the invitation. It was considered by the Eighth Congress, and formally accepted.

After the return of the committee to the United States, preliminary arrangements were begun by the committee of invitation, that, under the resolutions above given, was made "an Executive Committee with full power to fix the time and to make all necessary and suitable arrangements for the meeting of such congress, and to solicit funds for this purpose," and, "that the committee shall have power to add to its membership, to perfect its organization."

It seems that the committee, thus made an executive committee, considered that under the resolutions which authorized its appointment full power was conferred upon it to make *all* arrangements for the congress, even to the selection of the general officers and the arranging of the different working sections, and the completion of the organization of each section. In fact, that plenary power had been conferred upon the committee by those resolutions, and that the duties of the American Medical Association, practically, ceased with the appointment of the committee, and the appropriation of funds for the specified purposes.

A large part of the American Medical Association, who participated in the Washington meeting and voted for the resolutions, claims to have understood their import differently from the construction put upon them by the committee, which is now generally designated as "the original committee." The incompleteness of the instructions given by these resolutions, and the difference in the interpretation of them, seems to have been the starting-point of the differences which have so greatly divided the profession of our country, and caused so much misunderstanding and anxiety abroad. "The original committee" added to its number as it had been specially authorized to do, and proceeded not only "to perfect its organization," but also "to perfect" the organization of the congress.

The resolutions did not appoint the original committee to the offices of the congress, nor, on the other hand, did they say that the members of that committee should not be appointed officers of the congress. It is assumed that the members of the original committee, and those who were added to that committee, making the "General Committee," acted in accordance with what was believed to be for the best interest of the congress, although it has been charged that personal considerations had not, in some instances, been subordinated to the welfare of the congress. However, in discharging the important and difficult duty assigned the committee, it was not to be expected that it was practicable to wholly avoid mistakes, and in discharging its duties, it performed them very well, in many respects. The selections made were, in the main, men who are eminent, capable, and representative. Whilst it is claimed that mistakes were made, yet they were not such as would have had a specially unfavorable influence on the congress, except in the rules adopted regulating the American membership of the congress, which restricted the membership to delegates from organized regular medical societies and associations, "and of such persons as may be specially designated by the executive committee . . ." Such restriction had not been imposed at the meeting of a congress yet held.

Just prior to the annual meeting, in April, 1885, of the American Medical Association, in New Orleans, the Executive Committee announced the "Rules and Preliminary Organization" of the Ninth Congress, without having reported officially to the American Medical Association the fact that it had performed the duty assigned it, by the association, of presenting the invitation, or the fact that the invitation had been accepted.

These acts of omission, and of commission, gave rise to criticism upon the course of the Executive Committee, and to charges that it had exceeded its authority. There were those who, whilst admitting that the resolutions authorized the Committee of Invitation to elect *its* own officers, and to add to *its* membership, yet claimed that in saying that "it shall proceed to act as an Executive Committee, with full power to fix the time and to make all necessary and suitable arrangements for the meeting of such congress, . . ." authority was not thereby given to it to select the officers for the congress. It was further claimed that the committee which had now become the "Executive Committee" should have reported to the American Medical Association, from which it had received its appointment and its powers, the preliminary work that had been done, in arranging for the congress during the interval between the two annual meetings of the Association, and before making a public announcement of that preliminary work. It was further claimed that when such report should be made, by the Executive Committee, it was competent for the association—the appointing power—to receive such report and to retain, or alter, or discharge its committee, and to adopt, or modify, or reject the report of the work of its committee.

Here the second misunderstanding seems to have arisen. The Executive Committee claimed that it had not exceeded its authority, according to its understanding of the power conferred upon it by the resolutions adopted at the previous annual meeting of the association; that it had acted in good faith, and that it believed that its work had been well done, and done with an understanding, on its part, that the committee was to be continued intact, and that its work was not to be subject to revision or alteration by the association.

After discussion of these matters in the annual meeting of the American Medical Association in April, 1885, at New Orleans, when the "Original Enlarged Committee" made its report to the Association, it was

1. "*Resolved*, That the Committee appointed by this Association, to arrange for the meeting of the International Medical Congress in America, in 1887, be enlarged by the addition of thirty-eight members, one from each State and Territory, the District of Columbia, the Army, Navy and Marine Hospital service, to be appointed by the Chairman at this meeting, and that the committee, thus enlarged, shall proceed at once to review, alter and amend the motions of the present Committee as it may deem best."

(This resolution was amended by the provision, "that the members of the Committee should be selected by the respective State delegations.")

The following is a list of the Committee enlarged in accordance with the resolution as amended:

W. E. Anthony, M. D., Providence, R. I.; G. Baird, M. D., Wheeling, W. Va.; Robert Battey, M. D., Rome, Ga.; F. W. Beard, M. D., Vincennes, Ind.; J. S. Billings, M. D., U. S. Army, Washington, D. C.; J. M. Browne, M. D., U. S. Navy,

Washington, D. C.; L. P. Bush, M. D., Wilmington, Del.; H. F. Campbell, M. D., Augusta, Ga.; R. Beverly Cole, M. D., San Francisco, Cal.; E. P. Cook, M. D., Mendota, Ill.; W. C. Dabney, M. D., Charlottesville, Va.; Charles Denison, M. D., Denver, Col.; W. E. Duncan, M. D., Ellendale, Dakota Ter.; J. W. Dupree, M. D., Baton Rouge, La.; Ellsworth Eliot, M. D., New York City; G. J. Englemann, M. D., St. Louis, Mo.; N. F. Essig, M. D., Plattsburg, Mo.; Austin Flint, M. D., LL. D., New York City; E. P. Frazer, M. D., Portland, Oregon; George F. French, M. D., Minneapolis, Minn.; A. Y. P. Garnett, M. D., Washington, D. C.; S. C. Gordon, M. D., Portland, Me.; J. W. S. Gouley, M. D., New York City; F. M. Gunnell, M. D., U. S. Navy, Washington, D. C.; John B. Hamilton, M. D., U. S. Marine Hospital Service, Washington, D. C.; I. M. Hays, M. D., Philadelphia, Pa.; C. Johnston, M. D., Baltimore, Md.; George A. Ketchum. M. D., Mobile, Ala.; R. A. Kinloch, M. D., Charleston, S. C.; D. A. Linthicum, M.D., Helena, Ark.; John S. Lynch, M. D., Baltimore, Md.; J. J. McAchran, M. D., Laramie City, Wyom. Ter.; J. W. McLaughlin, M. D., Austin, Tex.; R. C. Moore, M. D., Omaha, Neb.; Robert Murray, M. D., U. S. Army, Washington, D. C.; R. D. Murray, M. D., Moultrie, Fla.; J. W. Parsons, M. D., Portsmouth, N. H.; William Pierson, M. D., Orange, N. J.; N. J. Pitman, M. D., Tarboro, N. C.; L. A. Sayre, M. D., New York City; X. C. Scott, M. D., Cleveland, O.; Nicholas Senn, M. D., Milwaukee, Wis.; John V. Shoemaker, M. D., Philadelphia, Pa.; F. L. Sim, M. D., Memphis, Tenn.; A. R. Smart, M. D., Hudson, Mich.; D. W. Stormont, M. D., Topeka, Kan.; J. M. Taylor, M. D., Corinth, Miss.; E. F. Upham, M. D., West Randolph, Vt.; W. H. Wathen, M. D., Louisville, Ky.; W. Watson, M. D., Dubuque, Iowa.; W. C. Wile, M. D., Sandy Hook, Conn.; A. H. Wilson, M. D., Boston, Mass.

At an informal meeting of the Committee, held at New Orleans during the session of the American Medical Association, in April, 1885, Dr. R. Beverly Cole, of San Francisco, Cal., was elected temporary Chairman, and Dr. John V. Shoemaker, of Philadelphia, Pa., was elected temporary Secretary.

Just before the final adjournment of the Association the following was adopted:

*Resolved*, That the Committee appointed in pursuance of a resolution adopted by this Association, April 30, 1885, to constitute an addition to the original Committee of seven previously appointed to invite and make arrangements for the meeting of the International Medical Congress, to be held in Washington, D. C., in 1887, be, and the said Committee is hereby, authorized and empowered to select a Chairman and a Secretary, and to fill all vacancies that may occur by death or inability to attend the Committee meetings, and to appoint the officers of the Congress.

This resolution has been construed as eliminating from the "General Committee" the President of the American Medical Association, added by vote, and, also, the members added by the "Original Committee," in accordance with the authority given it at the previous annual meeting of the Association. It is claimed that the wording of this resolution, whatever may have been its intent, is not conclusive that, by the passage of that resolution, the Association rescinded its act of a year before, when it authorized such additions to the membership of the "Original Committee." The two material points of this resolution are, that it is held by some that it left the status of the twenty-eight members added, in doubt; and that it definitely "empowered" its second "enlarged" Committee of Arrangements "to appoint the officers of the congress."

The second enlarged Committee held its first meeting at

Chicago, Ill., on June 24th and June 25th, 1885, for the purposes of organization and the transaction of the business committed to it by the American Medical Association.

In order to facilitate the holding of meetings in different sections of the country, the Committee deemed it advisable to select a Vice-Chairman, in addition to a Chairman and a Secretary.

The following named members were present at the meeting held in Chicago:

G. Baird, M. D., Wheeling, W. Va.
Robert Battey, M. D., Rome, Ga.
F. W. Beard, M. D., Vincennes, Ind.
J. S. Billings, M. D., Washington, D. C.
R. Beverly Cole, M. D., San Francisco, Cal.
E. P. Cook, M. D., Mendota, Ill.
W. E. Duncan, M. D., Ellendale, Dakota Ter.
Ellsworth Eliot, M. D., New York City.
N. F. Essig, M. D., Plattsburg, Mo.
G. F. French, M. D., Minneapolis, Minn.
A. Y. P. Garnett, M. D., Washington, D. C.
John B. Hamilton, M. D., Washington, D. C.
I. M. Hays, M. D., Philadelphia, Pa.
George A. Ketchum, M. D., Mobile, Alabama.
D. A. Linthicum, M. D., Helena, Ark.
John S. Lynch, M. D., Baltimore, Md.
J. W. McLaughlin, M. D., Austin, Texas.
X. C. Scott, M. D., Cleveland, O.
Nicholas Senn, M. D., Milwaukee, Wis.
John V. Shoemaker, M. D., Philadelphia, Pa.
F. L. Sims, M. D., Memphis, Tenn.
A. R. Smart, M. D., Hudson, Mich.
D. W. Stormont, M. D., Topeka, Kan.

E. F. Upham, M. D., West Randolph, N. Y.

W. H. Wathen, M. D., Louisville, Ky.

W. Watson, M. D., Dubuque, Iowa.

A. H. Wilson, M. D., Boston, Mass.

The resignation of Dr. Austin Flint, of New York, as a member of the Committee, was presented and accepted. Dr. J. W. S. Gouley, New York, was elected to fill the vacancy, and took his seat with the Committe.

The Committee then organized, a majority of its members being present, by the election of the following officers:

*Chairman*, Dr. R. Beverly Cole, San Francisco, Cal.

*Vice-Chairman*, Dr. John S. Lynch, Baltimore, Md.

*Secretary*, Dr. John V. Shoemaker, Philadelphia, Pa.

After the organization of the Committee, the number of members necessary for a quorum for future meetings was fixed at fifteen.

The following preamble and resolution were adopted, to apply to future meetings of the Committee:

" WHEREAS.—It is expedient that the meetings of this Committee shall represent, as far as practicable, the profession of all portions of our country,

" *Resolved.*—That any member of this Committee who may be unable to attend a meeting, shall be empowered to send as his proxy for the meeting any member of the American Medical Association, in good professional standing and a resident of his State or a member of his Government Department."

It was agreed that members of the Committee of Arrangements should not be elected officers of the Congress.

Under the instructions embodied in the Association's resolution, the committee proceeded to " review, alter and amend the action of the original committee of eight, by the adoption of the following general plan of organization.

1. The Congress shall be composed of members of the regular medical profession, who shall have inscribed their names on the register, and taken out their tickets of admission.

The American members of the Congress shall consist of delegates from the American Medical Association, and the medical societies in affiliation with the American Medical Association, each of said societies being entitled to appoint one delegate for every ten of its members.

The members of all special and subordinate committees, appointed by the Committee of Arrangements, shall also be entitled to membership in the Congress, and such other scieutific men as are approved by the Executive Committee,

All societies entitled to representation are requested to elect their delegates at their last regular meeting preceding the meeting of the Congress, and to furnish the Secretary-General with a certified list of the delegates so appointed.

2. The work of the Congress shall be divided into sixteen sections, as follows, namely:

1. Medical Education, Legislation and Registration, including methods of teaching, and buildings, apparatus, etc., connected therewith.
2. Anatomy.
3. Physiology.
4. Pathology.
5. Medicine.
6. Surgery.
7. Obstetrics and Gynæcology.
8. Ophthalmology.
9. Otology.
10. Dermatology and Syphilis.
11. Laryngology.
12. Public and International Hygiene.
13. Collective Investigation, Nomenclature, Vital Statistics and Climatology.

14. Military and Naval Surgery and Medicine.

15. Practical and Experimental Therapeutics.

16. Diseases of Children.

3. The general meetings shall be reserved for the transaction of the general business of the Congress, and for addresses or ommunications of scientific interest more general in character than those presented in the Sections.

4. Questions which have been agreed upon for discussion in the Sections shall be introduced by members previously nominated by the officers of the Sections. The members who may be appointed to open the discussions shall present, in advance, a statement of the conclusions which they have formed as a basis for debate.

5. Notices of papers to be read in any of the Sections, together with the abstracts of the same, must be sent to the Secretary of that Section before April 30, 1887. These abstracts will be regarded as confidential communications, and will not be published until the meeting of the Congress. Papers relating to questions not included in the list of subjects suggested by the officers of the various Sections will be received. Any member, after April 30, wishing to bring forward a subject not upon the programme, must give notice of his intention to the Secretary-General at least twenty-one days before the opening of the Congress. The titular officers and Council of each Section shall decide as to the acceptance of any communication offered to their Section, and shall fix the time of its presentation. No communication will be received which has been already published or read before a Society.

6. All addresses, papers and discussions made either at general meetings or in the Sections are to be immediately handed to the Secretaries. The Executive Committee, after the conclusion of the Congress, shall proceed with the publication of the Transactions, and shall have full power to decide which papers shall be published, and whether in whole or in part.

7. The official languages are English, French and German.

In the Sections no speaker will be allowed more than ten minutes, with the exception of readers of papers and those who introduce debates, who may occupy twenty minutes.

8. The rules, programmes and abstracts of papers shall be published in English, French and German.

Each paper or address will appear in the Transactions in the language in which it was delivered by the author. The debates will be printed in English.

9. The officers of the Committee of Arrangements shall be a Chairman, Vice-Chairman and a Secretary.

10. There shall be an Executive Committe, to be composed of the Chairman, Vice-Chairman, Secretary-General, Treasurer, the Chairman of the Finance Committee, the Secretary and five other members, to be elected by the Committee of Arrangements. The duties of the Executive Committee shall be to carry out the directions of the Committee of Arrangements, to authorize such expenditures as may be necessary, and to act for the Committee during the intervals of its sessions, reporting such action at the next meeting of the Committee, for approval.

11. There shall be a Standing Committee on Finance, composed of one person from each State, Territory, the District of Columbia, the Medical Departments of the Army, Navy and the Marine Hospital Service. The Chairman of the Finance Committee shall be *ex officio* one of the Vice-Presidents of the Congress, and also a member of the Executive Committee. The Committee of Arrangements shall appoint the Finance Committee, and each State chairman appoint the local Finance Committee of one from each Congressional District.

12. The officers of the Congress shall be a President, such number of Vice-Presidents as may hereafter be determined on, a Secretary-General, two Assistant Secretaries, also to be hereafter appointed, and a Treasurer, and those elected to these positions will be nominated by the Committee of Arment, to hold the same offices in the Congress.

Dr. Austin Flint was continued as President of the congress, as were many of the other officers previously selected. In fact, the personel of the congress was not greatly changed. Two of the Vice-Presidents were not continued, and eleven others were added. The Secretary-General declined to be continued, and another was elected to that office.

The number of the sections was reduced from nineteen to sixteen, by merging sections. Twelve of the Presidents of sections remained unchanged. The merging of sections changed three of the presidents, and four others were not continued.

In the offices of Vice-President, Secretary and Council, the principal changes made were in enlarging the number of each.

The principal changes made in the rules for the government of the congress consisted in requiring that "the American members of the congress shall consist of delegates from the American Medical Association, and the medical societies in affiliation with the American Medical Association," which restricted the American membership even more than the conditions imposed by the "original enlarged committee," and made the same mistake of proposing two classes of members for the congress. Another change reduced the number of the sections, and, consequently, of presidents of sections, as previously stated. But little change was made regarding the organization and the work of the sections.

Having progressed thus far toward the organization of the congress, the Committee of Arrangements adjourned.

After the adjournment it was charged that the action taken was partisan, hasty and ill-advised, and in contravention of the best interests of the congress, for which alleged reasons numerous resignations from the organization occurred.

---

The second meeting of the Committee of Arrangements was held in New York city, on the 3rd of September. The following named members were present:

Dr. G. Baird, Dr. Robert Battey, Dr. L. P. Bush, Dr. R. Beverly Cole, Dr. W. C. Dabney, Dr. Ellsworth Eliot, Dr. A. Y. P. Garnett, Dr. S. C. Gordon, Dr. J. W. S. Gouley, Dr. J. B. Hamilton, Dr. George A. Ketchum, Dr. R. A. Kinloch, Dr. D. A. Linthicum, Dr. John S. Lynch, Dr. R. C. Moore, Dr. William Pierson, Dr. N. J. Pitman, Dr. L. A. Sayre, Dr. X. C. Scott, Dr. John V. Shoemaker, Dr. F. L. Sim, Dr. E. F. Upham, Dr. W. H. Wathen, Dr. W. C. Wile, Dr. A. H. Wilson.

Other members were represented by proxies:

Dr. E. P. Cook, by Dr. N. S. Davis, proxy; Dr. A. R. Smart, by Dr. William Brodie, proxy; Dr. J. M. Taylor, by Dr. E. P. Sale, proxy.

The committee was called to order September 3rd, 1885, by the Chairman, Dr. R. Beverly Cole.

The resignation of Dr. L. A. Sayre, of New York, as member of the committee was tendered in consequence of his ill-health. It was accepted, and Dr. A. Flint, Jr., of New York, was elected to fill the vacancy and took his seat with the committee.

There were seven vacancies in the Committee of Arrangements, and the following gentlemen were elected to fill their vacancies:

Dr. J. Bartlett, Wisconsin; Dr. J. H. Baxter, U. S. Army; Dr. George Goodfellow, Arizona; Dr. Henry Leffman, Pennsylvania; Dr. John Morris, Maryland; Dr. J. R. Tipton, New Mexico; Dr. Thomas J. Turner, U. S. Navy.

It was decided that no person should occupy more than one position in the organization of the congress.

It was also decided that, in the published lists of the officers of the congress, the names of the Vice-Presidents and Secretaries of the congress, and the Vice-Presidents, Secretaries and members of Councils of the Sections, should be arranged alphabetically.

The Section of Medical Education was discontinued. The Sections of Gynæcology and Psychological Medicine and Diseases of the Nervous System, and of Dental and Oral Surgery were restored. The Section of Laryngology was united with that of Otology.

In the interval between the first meeting of the committee in Chicago, in June, and the second meeting in New York, in September, it had become evident that it was not best to make two classes of members of the congress, and that no effort should be made to make the congress a delegated body. Therefore the Committee of Arrangements wisely concluded to remove all previous restrictions imposed, and to make the requirements for membership as liberal as they had been in any congress yet held, and adopted the following plan of organization:

THE RULES ADOPTED ARE:

1. The congress shall consist of members of the regular profession of medicine, who shall have inscribed their names on the register and shall have taken out their tickets of admission; and of such other scientific men as the Executive Committee of the congress may see fit to admit.

2. The dues for members of the congress shall be ten dollars each for members residing in the United States.

There shall be no dues for members residing in foreign countries.

Each member of the congress shall be entitled to receive a copy of the "Transactions" for 1887.

3. The congress shall be divided as follows, into seventeen sections:

I. General Medicine.

II. General Surgery.

III. Military and Naval Surgery.

IV. Obstetrics.

V. Gynæcology.

VI. Therapeutics and Materia Medica.

VII. Anatomy.

VIII. Physiology.

IX. Pathology.

X. Diseases of Children.

XI. Ophthalmology.

XII. Otology and Laryngology.

XIII. Dermatology and Syphilis.

XIV. Public and International Hygiene.

XV. Collective Investigation, Nomenclature, Vital Statistics, and Climatology.

XVI. Psycological Medicine and Diseases of the Nervous System.

XVII. Dental and Oral Surgery.

4. The general meetings of the congress shall be for the transaction of business and for addresses and communications of general scientific interest.

5. Questions and topics that have been agreed upon for discussion in the sections shall be introduced by members previously designated by the titular officers of each section. Members who shall have been appointed to open discussions shall present in advance statements of the conclusions which they have formed as a basis for debate.

6. Brief abstracts of papers to be read in the sections shall be sent to the secretaries of the proper sections on or before April 30, 1887. These abstracts shall be treated as confidential communications, and shall not be published before the meeting of the congress.

Papers relating to topics not included in the lists of subjects proposed by the officers of the sections may be accepted after April 30, 1887; and any member wishing to introduce a topic not on the regular lists of subjects for discussion shall give notice of the same to the secretary-general, at least twenty-one days before the opening of the congress, and such notices shall be promp ly transmitted by the secretary-general to the presidents of the proper sections. The titular officers of each section shall decide as to the acceptance of such proposed communications and the time for their presentation.

7. All formal addresses, scientific communications and papers presented, and scientific discussions held at the general meetings of the congress, shall be promptly given in writing to the secretary-general; and all papers presented and discussions held at the meetings of the sections shall be promptly given in writing to the secretaries of the proper sections.

No communication shall be received which has already been published, or read before a society.

The Executive Committee, after the final adjournment of the congress, shall direct the editing and the publication of its "Transactions," and shall have the full power to publish the papers presented and the discussions held thereon, either in full, in part, or in abstract, as in the judgment of the committee may be deemed best.

8. The official languages of the congress shall be English, French and German.

In the meetings of the sections, no member shall be allowed to speak for more than ten minutes, with the exceptions of the readers of papers and those who introduce subjects for discussion, who may each occupy twenty minutes.

9. The rules and programmes shall be published in English, French and German.

Each paper and address shall be printed in the "Transactions" in the language in which it was presented, and preliminary abstracts of papers and addresses also shall be printed, each in the language in which it is to be delivered.

All discussions shall be printed in English.

10. The president of the congress, the secretary-general, the treasurer, the chairman of the Finance Committee, and the presidents of the sections, shall together constitute an Executive Committee of the congress, which committee shall direct the business of the congress, shall authorize all expenditures for the immediate purposes of the congress, shall supervise and audit the accounts of the treasurer, and shall fill all vacancies in the offices of the congress and of the sections. This committee shall have power to add to its membership, but the total number of members shall not exceed thirty. A number equal to one-third of the members of the committee shall constitute a quorum for the transaction of business.

11. The officers of the congress shall be a president, vice-presidents, a secretary-general, four associate secretaries, one of whom shall be the French secretary, and one of whom shall be the German secretary, a treasurer, and the chairman of the Finance Committee.

12. The officers of each section shall be a president, vice-presidents, secretaries, and a council.

13. The officers of the congress and the officers of the sections shall be nominated to the congress at the opening of its first session.

14. The Executive Committee shall, at some convenient time before the meeting of the congress, prepare a list of foreign vice-presidents of the congress and foreign vice-presidents of the sections, to be nominated to the congress at the opening of its first session.

15. There shall be a standing Committee on Finance, composed of one representative from each state and territory, the District of Columbia, the medical department of the army, the medical department of the navy, and the marine hospital service.

The chairman of the Finance Committee shall report to the Executive Committee of the congress.

Each member of the Finance Committee shall appoint a local Finance Committee for his state, territory, district, or government department, consisting of one or more members from each government department or congressional district.

Each local Finance Committee shall report through its chairman to the chairman of the Finance Committee of the congress.

The committee then proceeded to fill the more important positions found to be vacant in the organization of the congress. The following is a correct list of the general officers of the congress, including Vice-Presidents, and also the Presidents of the seventeen sections:

PRESIDENT.

Austin Flint, M. D., LL. D., New York.

VICE-PRESIDENTS.

W. O. Baldwin, M. D., Alabama; *H. I. Bowditch, M. D., Massachusetts; William Brodie, M. D., Michigan; *Henry F. Campbell, M. D., Georgia; W. W. Dawson, M. D., Ohio; *R. Palmer Howard, M. D., Canada; E. M. Moore, M. D., New York; Tobias G. Richardson, M. D., Lousiania; Lewis A. Sayre, M. D., New York; J. M. Toner, M. D., District of Columbia; the President of the American Medical Association; the Surgeon-General of the United States Army; the Surgeon-General of the United States Navy; the Supervising Surgeon-General of the United States Marine Hospital Service.

SECRETARY-GENERAL.

Nathan S. Davis, M. D., LL. D., Chicago, Illinois.

TREASURER.

E. S. F. Arnold, M. D., M. R. C. S., New York.

CHAIRMAN OF THE FINANCE COMMITTEE.

Frederick S. Dennis, M. D., M. R. C. S., New York.

EXECUTIVE COMMITTEE OF THE CONGRESS.

GENERAL OFFICERS.

Austin Flint, M. D., LL. D., President of the Congress.

Nathan S. Davis, M. D., LL. D., Secretary-General.

E. S. F. Arnold, M. D., M. R. C. S., Treasurer.

Frederick S. Dennis, M. D., M. R. C. S., Chairman of the Finance Committee.

PRESIDENTS OF THE SECTIONS.

*General Medicine.*—Abram B. Arnold, M. D.

*General Surgery.*—William T. Briggs, M. D.

---

*Since resigned.

*Military and Naval Medicine and Surgery.*—Henry H. Smith, M. D.

*Obstetrics*—DeLaskie Miller, M. D., Ph. D.

**Gynæcology.*—Robert Battey, M. D.

*Therapeutics and Materia Medica.*—F. H. Terrill, M. D.

*Anatomy.*—William H. Pancoast, M. D.

**Physiology.*—John C. Dalton, M. D.

**Pathology.*—E. O. Shakespeare, M. D.

*Diseases of Children.*—J. Lewis Smith, M. D.

*Ophthalmology.*—A. W. Calhoun, M. D.

*Otology and Laryngology.*—S. J. Jones, M. D., LL. D.

*Dermatology and Syphilis.*—A. R. Robinson, M. D.

*Public and International Hygiene.*—Joseph Jones, M. D.

*Collective Investigation, Vital Statistics, and Climatology.*—Henry O. Marcy, M. D.

*Psychological Medicine and Nervous Diseases.*—John P. Gray, M. D., LL., D.

*Dental and Oral Surgery.*—Jonathan Tafft, M. D.

### LOCAL COMMITTEE OF ARRANGEMENTS.

(With power to increase the number.)

A. Y. P. Garnett, M. D., Chairman, Dist. of Columbia.

The Surgeon-General U. S. Army.

The Surgeon-General U. S. Navy.

The Supervising Surgeon-General U. S. Marine Hospital Service.

J. H. Baxter, M. D., District of Columbia.

C. H. A. Kleinschmidt, M. D., District of Columbia.

N. S. Lincoln, M. D., District of Columbia.

J. M. Toner, M. D., District of Columbia.

---

*Since resigned.

Lists of Vice-Presidents, Secretaries, and Councilmen for each section were named by the Committee of Arrangements, but as it was not practicable to ascertain at once who would accept the places assigned them, or who of those who had been announced in the medical press as declining to accept positions before the present rules and organization had been adopted as given above might wish to withdraw such declination, the final adjustment of these offices was referred to the Executive Committee of the congress, and all correspondence in relation thereto was transferred to the Secretary-General of the congress.

Having thus selected the officers of the congress and the presidents of the sections, who together were made an "Executive Committee," the committee of arrangements considered its work practically completed, and placed the affairs of the congress in charge of the "Executive Committee," and now awaits the assembling of the American Medical Association in St. Louis, in May next, to report the completion of the work assigned to it by the Association, whereupon the Association will be in position to say to the medical profession of the country that it discharged the duty undertaken by it, of extending an invitation to have the ninth congress held in Washington; that the invitation was accepted and that, through its committee of arrangements, the congress has been organized and the affairs of the congress placed in charge of the executive committee of the congress, and that the Association need no longer regard itself as holding any official relation to the congress.

It was with this understanding of its status that the Executive Committee assumed the management of the work of the congress, and it so announced after its first meeting and organization.

The first meeting of the executive committee of the congress occured in New York city on September 24th, and was organized by electing Henry H. Smith, M. D., Chairman. The office of Associate Secretary-General was created, and F. S. Dennis, M. D., was elected to that office, and R. J. Dunglison, M. D., was elected chairman of the finance committee, in place of F. S. Dennis, transferred.

After the transaction of some routine business, and directing that an official announcement be made that the preliminary organization of the congress had been effected, the committee adjourned.

The second meeting of the executive committee occured in New York city on November 18th. There being vacancies in the office of President of three of the sections, the executive committee numbered nineteen; of these, fifteen were present.

R. J. Dunglison, M. D., was elected permanent Secretary of the committee. Some routine business was transacted, and some vacancies in some of the sections were filled.

Then, acting under the authority conferred upon the executive committee by rule ten, and as contemplated by the committee of arrangements when it transferred the affairs of the congress to the executive committee, the latter committee unanimously elected six additional members of the committee, viz.:

J. S. Billings, M. D., U. S. Army; J. M. Browne, M. D., U. S. Navy; Christopher Johnston, M. D., of Baltimore, and G. J. Engleman, M. D., of St. Louis, all of the "original committee", and, also, William Pepper, M. D., and J. M. Da Costa, M. D., both of Philadelphia.

It was then decided to postpone the filling of the other vacancies, including the presidencies of the three sections, in order that the newly-elected members might participate in filling them, if they should accept the positions to which they had been elected.

This course on the part of the committee was taken in recognition of the fact that there exists much misunderstanding in the profession regarding the organization of the congress, which fact has prevented the cordial coöperation ot many whose recognized rank in the medical profession entitles them to prominent positions in a congress, international and important in character. The committee appreciated the fact that it now stands in a capacity of trust, not alone for the medical profession of the United States, much less for fac_tions in it, but for the entire medical profession that is expected to participate in the congress, and that its course of action should be such as would give evidence that it appreciated the fact and felt the responsibility that attaches to such an important trust. It therefore sought, in making selection of those to be added, to elect such as would properly represent those not represented in the preliminary organization. Having done this, the committee adjourned, and awaits the action of the newly-elected members.

